# Full-mouth rehabilitation of an acromegaly disease patient with removable prostheses: a clinical case report

**DOI:** 10.11604/pamj.2019.33.5.18194

**Published:** 2019-05-06

**Authors:** Sana Bekri, Wijdène Trifi, Amel Labidi, Chaima Bizani, Lamia Mansour

**Affiliations:** 1 Department of Removable Prosthodontics, Faculty of Dental Medicine of Monastir, Tunisia

**Keywords:** Acromegaly, rehabilitation, removable prosthodontics

## Abstract

Acromegaly is a rare disease that can have serious consequences. This disease is not widely known by stomatologists, although cranio-facial manifestations are important and rapid changes in the oral cavity are frequent and sometimes dramatic. The prosthetic management of patients with acromegaly is not easy because it is necessary to wait for the stabilization of the disease. If this is not the case, the bone changes prevent the stability of prostheses over time. Moreover, because of bone remodeling, some treatments are contra-indicated, which limits our therapeutic choices. Through this clinical case, we will focus on the oral manifestations of patients with acromegaly and the method of prosthetic management.

## Introduction

Acromegaly is a rare endocrine disease that was first medically described by Nicolas Saucerotte in 1772 [1]. Then, in 1864, acromegaly was described under the name of prosopectasia by the Italian anatomist Andrea Verga. Later on, Pierre Marie in 1889 described the whole clinical picture [2]. This disease is characterized by a chronic and unrestrained hyper-secretion of the growth hormone produced by the pituitary gland after the epiphyseal plate closure at puberty. Its prevalence varies between 2.8 and 13.7 cases per 100 000 people with an annual incidence of 0.2 and 1.1 cases per 100 000 people [3]. Acromegaly is associated with increased morbidity and mortality as well as life quality's limits. Patients often have cardiovascular problems, diabetes mellitus, phosphocalcic disturbances, and lipid metabolism disorders [4]. On the other hand, this disease causes numerous complications as well as significant structural changes in the oral cavity [5]. The dysmorphic syndrome that characterizes acromegaly is dominated by an enlargement of the thorax and a projection of the sternum forward by the proliferation of costochondral joints, an enlargement of the hands and feet, a thickening of the lips and nose, a protrusion of the superciliary arches due to the pneumatization of the frontal sinuses, a mandibular prognathism and an opening of the gonion angle [5]. The dental complications are partially or completely irreversible after the correction of the excess secretion of the GH. This reinforces the idea of getting a diagnosis as soon as possible to avoid or at least to reduce the consequences of acromegaly .In dental literatures; there are very few precise descriptions of the odonto-stomatological and especially the prosthetic management of these patients. The aim of this article was to precise the different stomatological manifestations of acromegaly, and to highlight upon the problems encountered during the prosthetic rehabilitation of an acromegalic patient through a clinical case.

## Patient and observation

Mr. NA, 63 years old, with acromegaly, is followed in the endocrinology department of Charles Nicolle Hospital Tunis, Tunisia. This patient was referred to the Monastir Dental Clinic by his attending physician for a possible prosthetic rehabilitation. Its acromegaly was detected following facial dysmorphism with hypertrophy of the extremities. The diagnosis was made in 2001 following a somatotropic test value which showed an increase in the growth hormone GH (18ng/ml) and IGF-1 (825ng/ml). The Glucose test was abnormal: the lowest value was 3.56ng/ml. MRI revealed the existence of a pituitary macro-adenoma measuring 1.5cm. The patient reported that he also suffered from high a blood pressure and diabetes mellitus. Endoscopic transphenoidal surgery was upheld upon pituitary lodge's lesion. In 2016, hypersomatotropism was discovered by a high non-breakable GH value. Besides, a high IGF-1 level revealed a probable adenomatous residue for which the patient was treated with drugs such as LANREOTIDE (SOMATULINE^®^ (LP 90)) up to 2018.

**The extra-oral examinations:** focused on acromegaly's main characteristics such as: the imposing face, the enlargement of nasal base, the thickened lips, the salient features of cheekbones and borrow ridge. The profile view showed a mandibular prognathism and a procheilia of the lower lip (Figure 2), as well as hypertrophic masticatory mucles.

**Figure 1:**
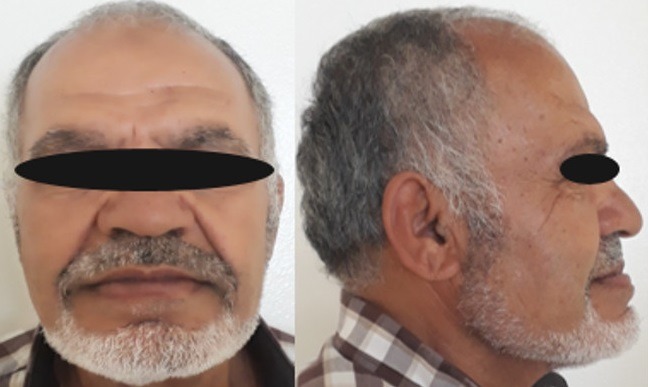
Extraoral examination

**Figure 2:**
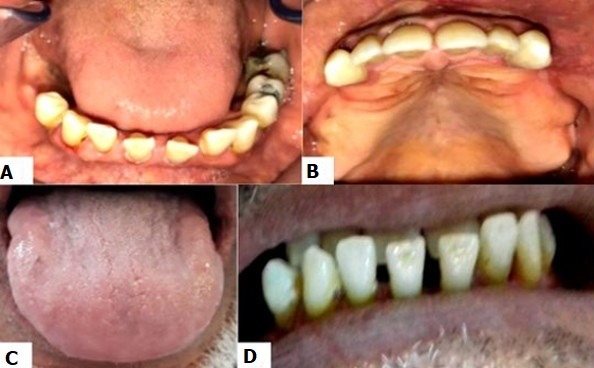
Intraoral examination; (A, B) arches enlargement; (C) macroglossia; (D) diastema

**Figure 3:**
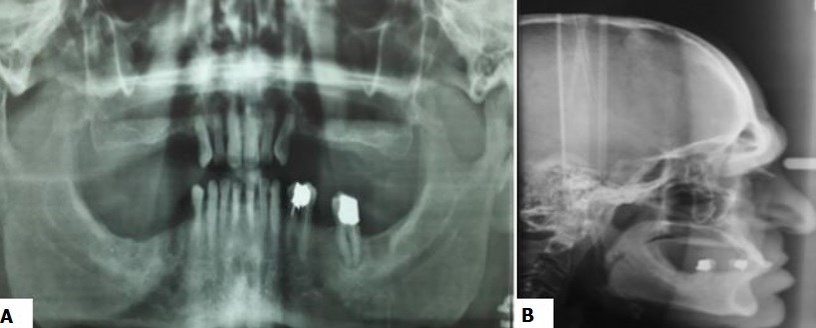
X-Ray examination; (A) orthopantomogram examination; (B) cephalogram examination

**Figure 4:**
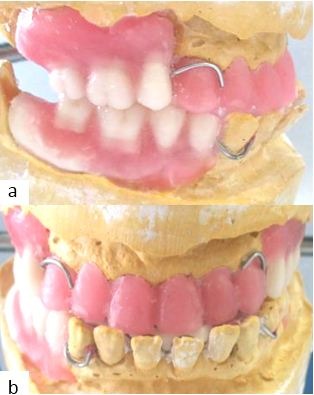
The prosthetic project: (A) lateral view; (B) anterior view

**The intraoral examination** showed the final stage of a bilateral edentulous maxillae and a final edentulism of the mandible bordered by the 44^th^ tooth (Figure 2A, Figure 2B). The enlargement of arches was found at mandibular level. At the maxillary level, the ridges were tall, well-formed and covered by a firm and adherent fibro-mucosa with a broad and moderately deep palate. At the mandibular level, the alveolar ridge was moderately high with a firm fibro-mucosa. A prominent macroglossia with the impression of the mandibular teeth on the anterior and lateral edges of the tongue was noted (Figure 2C). The residual mandibular teeth were separated by diastema. A generalized recession ranging from 2 to 4mm was found and a first degree of mobility was registered in all residual maxillary and mandibular teeth except the 44^th^ tooth (third degree mobility). The occlusal examination showed an anterior inverted occlusion (Figure 2D), a collapsed vertical dimension of occlusion and a lack of the anterior guidance. This clinical examination was completed by an X-ray examination (Figure 3A, Figure 3B); an orthopantomogram (OPG) and a cephalogram were requested. The OPG revealed a ratio radiological crown / radiological root equal to 1 in the maxillary teeth. This ratio was bigger than or equal to 1 in the mandibular teeth. The cephalometric analysis (Table 1) confirmed the presence of a skeletal class III associated with a hypo divergence. A widening of sella turcica was also noticed. The therapeutic goals were to improve the masticatory and phonetic functions, to establish the vertical dimension of occlusion (VDO) as well as the functional curves and to improve aesthetics. After confronting the clinical and radiological data and discussing the therapeutic goals with the patient, the treatment chosen in this case was maxillary combined prosthesis with a metal-ceramic fixed partial prosthesis (FPP) restoring the residual teeth and a metallic partial removable prosthesis (PRP) replacing the missing teeth. The retention means lead to choose two RPL clasps. The attachments were discarded for financial reasons. As for the mandibule, the decision was to make a transient resin- RPP. This prosthetic management was complex because of the prognathism and the inverted occlusion especially that the patient was keen to improve the state of anterior teeth. Therefore, set up diagnostic wax-up and diagnostic occlusal adjustment were made in order to establish accurate diagnosis and treatment planning (Figure 4A, Figure 4B). This facilitated the communication with the patient by the materialization of the treatment project and by the visualization of the final result that would had given the chosen management. This pre-assembly was also necessary to test the new VDO which has been raised by 6 mm at the incisors in order to improve the ratio of the anterior teeth. The facial typology and articulatory temporomandibular joint examination favored this increase of VDO.

**Table 1:** Cephalometric analysis

	Cephalometric measurements
SNA	81°
SNB	88°
ANB	-7°
I /F	120°
I /i	112°
FMA	12°
IMPA	137°
GOGN /SN	21°

**Figure 5:**
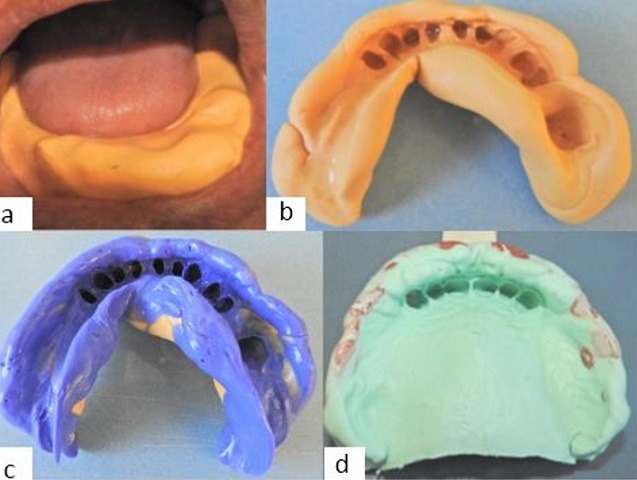
Preliminary impressions: (A, B, C) mandibular preliminary impression; (D) maxillary preliminary impression

**Prosthetic management:** preliminary impression: the excessive width of the mandibular arch was the first difficulty encountered. For this patient, all the impression trays that we had at our disposal were too small. The primary impression with high and low viscosity silicone using the flexible impression technique has been a reliable alternative to overcome this problem (Figure 5A, Figure 5B, Figure 5C). The consequences of acromegaly were less visible in the maxilla than in the mandible. Adjustments of the plastic trade tray were made by heating it slightly to spread its edges and then by rectifying it with wax (Figure 5D).

**Provisional FPP and RPP:** the peripheral preparation of the 13, 12, 11, 21, 22 and 23 was made in order to set ready the ceramic-metallic FPP. Furthermore, the patient has benefited of provisional prostheses which were used as a guide for the final FP. They have permitted to test the new VDO and solve the temporary aesthetic problem.

**Figure 6:**
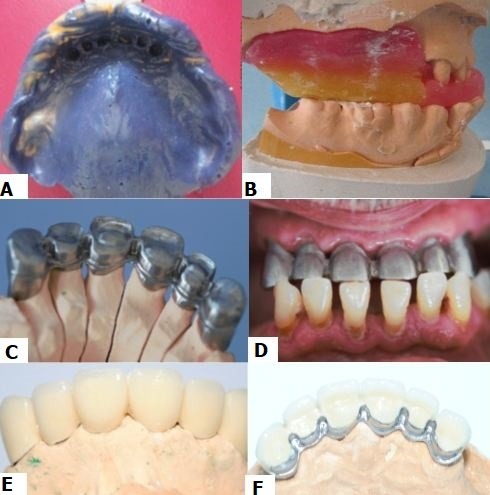
Realization of fixed partial prosthesis; (A) maxillary impression; (B) maxillo-mandibular record; (C, D) metal frame; (E) ceramic mounting; (F) frame millings

**Maxillary impression:** the maxillary impression was taken from silicones with high and low viscosities according to the Wash-technique without a particular precaution (Figure 6A).

**Occlusal registration:** the registration of occlusion for the FPP was made in the centric relationship according to the new VDO (Figure 6B) and sent to the lab with the corresponding metallic framework shema. The main maxillary connection was anteriorly and posteriorly cleavaged in order to alleviate the metallic framework. At the lab, the wax sculpture of the infrastructure was made. Millings and guidance plans were planned at this stage using the parallelizer. After FPP metal casting (Figure 6C), the metal frame was fitted inside the mouth (Figure 6 D). Thereafter, the technician made the assembly of the ceramic (Figure 6E).

**Figure 7:**
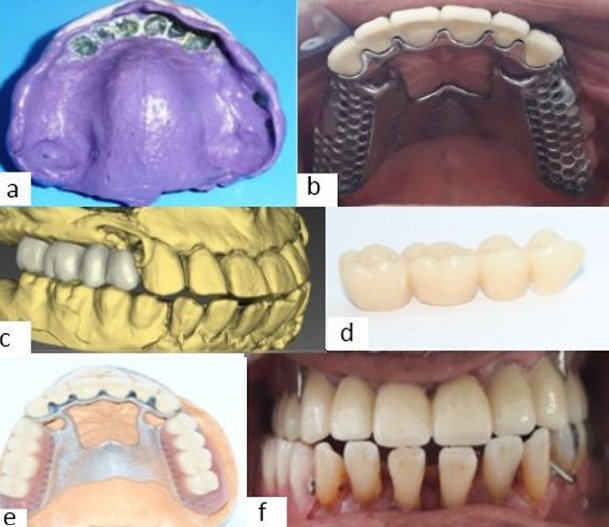
Removable partial prosthesis; (A) anatomic-functional impression; (B) metal framework fitting; (C, D) prosthetic teeth carving; (E) prosthetic teeth mounting; (F) combined prosthesis

**RPP making:** an anatomic-functional impression with a polyether was made (Figure 7A) to make the metal frame. After fitting the frame in mouth (Figure 7B), the occlusion was recorded for mounting the prosthetic teeth. This laboratory step was specifically delicate. Indeed, the choice of teeth had been important. We had to pick teeth a little wider than those available in dental market. As a result, the lab technician proceeded to CAD-CAM tooth carving (Figure 7C, Figure 7D) in order to better align the occlusal contacts with the opposing teeth. The posterior prosthetic teeth (Figure 7E) were mounted on the top of the ridge, the left-side teeth were in the occlusal norms, while on the right side the reports were crossed according to the large width of the mandibular arch. This assembly was tried and validated in mouth then polymerized. Prostheses were inserted in mouth and the occlusal equilibration was controlled during the session. Hygiene and maintenance advice were renewed. A follow-up appointment was set after one week and periodic checks were scheduled for possible adjustments.

## Discussion

The oral conditions induced by acromegaly had complicated the prosthetic management. Mandibular prognathism, macroglossia, diastema, and malocclusions had been described in the literature as frequent manifestations of acromegaly. The mandibular prognathism, observed in this patient, is often the most striking physical feature of acromegaly [6]. This prognathism results from the appositional growth and hypertrophy of the condylar cartilage, which is aggravated by the pressure exerted by the tongue. The study by Künzler *et al.*[7] showed that 16 to 57% of the acomegalic patients had a prognathism with an average protrusion of the mandible of 9.3 +/- 3.4mm. As for the maxillary bone, most authors agree that it does not undergo deformities in acromegaly [8]. Macroglossia is considered a possible cause of mandibular prognathism. Indeed, it has been suggested that an increase in soft tissue volume induces an osteogenic reaction in bone growth sites [6]. Macroglossia can cause difficulties with swallowing and articulation. The enlargement of the tongue may cause the anterior flaring of the teeth and the narrowing of the air space of the pharynx which may be the cause of the occurrence of frequent Obstructive Apnea Syndrome in the acromegalic patients. Class III occlusion is the most typical feature and its frequency is between 25% to 77% [7]. Hermann *et al* [9] found the anterior cross occlusion in 48% of cases and end-to-end occlusion in 56% of cases. The presence of diastema is observed in 93.75% of acromegalic patients, and is caused by an increase in the mandibular volume [10], pressure of the tongue and malocclusion. There is an increase in tooth mobility especially in the anterior teeth of the lower jaw [8, 10]. Dental mobility is caused by several factors: severe occlusal trauma due to class III malocclusions, excessive chewing force resulting from the exaggerated mandibular enlargement and muscular hypertrophy and the pressure of the tongue on teeth [10]. Acromegaly can also affect the temporomandibular joint; growth hormone promotes endochondral and periosteal bone growth. The growth of cartilage on the condyle may cause irregularities in shape. For acromegalic patients seeking prosthetic rehabilitations, the therapeutic decision depends on several clinical parameters, the benefit, the cost, the safety ratio and the feasibility of the treatments taken into consideration.

Ideally for this patient, it was necessary to consider an orthodontic-surgical treatment for the correction of the skeletal and the dental class III followed by a fixed or removable implant-supported prosthetic rehabilitation. These interventions should only be considered when growth hormone levels have been stabilized. In this case, the patient had not yet had a good regulation of GH secretion. Thus, the choice of one of these proposals was not retained because of the significant bone remodeling. The complexity of the treatment was due to the absence of the orthodontic-surgical preparation. Therefore, an increase of the vertical dimension of occlusion was made in order to compensate the skeletal class III and to improve the facial aesthetics. However, it is important to remember that the increase in OVD is not systematic and that there are several other decision indicators other than skeletal typology. It is imperative to consider the temporo-mandibular joint which could be the seat of morphological changes due to the dysmorphic syndrome in acromegaly, the neuromuscular coordination, the aesthetics (a patient with acromegaly disease has a long side which counter-indicates the increase of the VDO), the bilabial contact and the relationship of the anterior teeth (occlusal indicator) and the anterior and posterior prosthetic height (prosthetic indicator). The class III Angle and the mandibular prognathism are also problematic when mounting the prosthetic teeth and they affect the aesthetic and functional outcomes. It should be noted that trying to mount the teeth on the ridge is not very recommendable. In fact, the space left to the tongue is considerably reduced by the exaggerated lingual and palatal positioning of the teeth, causing a constant instability of the removable prostheses. An inverted occlusion is most often performed. This type of assembly reserves the needed space for the tongue. However, it often leads to poorly controlled premature contact. Balancing in deduction is difficult and cheek bites will be common. In addition, interferences on the non-working side cause and amplifie bone resorption phenomena. To cope with the problem found during the assembly of the prosthetic teeth, it was more judicious to set up the dental units in the prosthetic corridor recorded with piezography for a better compromise of efficiency and comfort.

## Conclusion

The mouth is the seat of many anomalies' manifestations, including acromegaly. These anomalies can lead dentists to become actors for earlier detection of the disease. Odontological management of acromegaly requires a careful work by dentists who must be aware of the existence of this rare disease. Dentists' collaboration with other medical specialties will definitely optimize the longevity of the results obtained by the established therapy.

## Competing interests

The authors declare no competing interests.
